# Moderate Processing of Rice: Processing Technology, Quality Evaluation, and Reflections Precision

**DOI:** 10.3390/foods15152696

**Published:** 2026-07-30

**Authors:** Zeren Wang, Changyuan Wang, Shan Zhang, Hongchen Ren, Chuanying Ren

**Affiliations:** 1 College of Food Science, HeilongjiangBayi Agricultural University, Daqing 163000, China; 19990716999@163.com; 2 Food Processing Research Institute, Heilongjiang Academy of Agricultural Sciences, Harbin 150086, China; zhangshanfood@163.com; 3 Heilongjiang Academy of Agricultural Machinery Engineering Sciences, Heilongjiang Academy of Agricultural Sciences, Harbin 150086, China; renhongchenzaici@163.com

**Keywords:** moderate processing of rice, save food, biological activity, intelligent rice milling, nutrient retention

## Abstract

Accurate and moderate rice processing is a key strategy for ensuring national food security and improving population nutritional status. This article synthesizes the technical scope and development trajectory of moderate processing, examines the evolution from multistage light grinding to AI-driven targeted grinding, and analyzes the balance between processing accuracy and eating quality, nutritional retention, storage stability, and safety risks. Evidence suggests that the core of moderate processing lies in the dynamic alignment of rice variety with processing targets, supported by intelligent online detection technologies that enable precise control. China’s rice processing industry is currently transitioning from experience-driven to data-driven operations; however, it continues to face challenges, including underdeveloped evaluation standards, high technology adoption costs, and limited consumer awareness. Future efforts should prioritize loss reduction, nutritional preservation, and intelligent processing to support coordinated advancement across the precision and moderate rice processing value chain. Moderate processing of rice preserves more native nutrients and plays an important role in improving residents’ dietary structure and safeguarding public health, achieving the best balance between saving food, preserving nutrients, and improving efficiency.

## 1. Introduction

**Figure 1:**
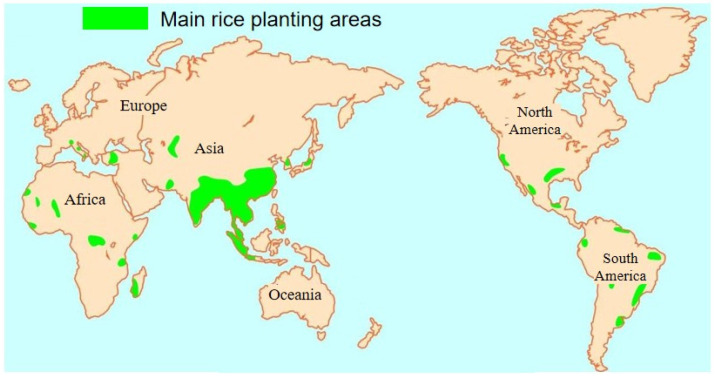
Schematic diagram of world rice distribution. The world’s rice cultivation is concentrated in Asia, Europe, Africa, North America, and South America, with Asia having the largest planting area and accounting for over 90% of the world’s rice production.

Rice is the staple food for over half of the global population. A total of 122 countries cultivate rice across approximately 165 million ha. Mainly concentrated in Asia, Europe, Africa, North America, and South America, with Asia having the largest planting area and accounting for 90% of the world’s rice production. (Figure 1), with an annual production of approximately 755–780 million tons. Asia dominates global rice production, contributing over 90% of total output, with major producers including India, China, Indonesia, Bangladesh, and Vietnam. India and China have the largest cultivation areas, accounting for 28.1% and 18.5% of the global rice area, respectively, and contributing 21.7% and 28.5% of total rice production, respectively. China is the world’s leading rice producer. By 2050, the global population is projected to reach 9.8 billion, while per capita arable land is expected to decline from 0.45 ha to 0.21 ha [1]. These trends underscore the urgency of strengthening food security through sustained efforts to increase agricultural productivity and efficiency, including the strategic integration of land and technological innovations to support a more efficient, high-quality, and sustainable food system.

Major rice losses occur during harvesting, storage, processing, and consumption. Processing is particularly critical, involving up to 28 steps from paddy to milled rice, including repeated milling and polishing. Each additional milling or polishing step increases grain loss, raises the proportion of broken rice, elevates energy consumption, and reduces overall yield [2]. For example, increasing the degree of milling (DOM) from 5% to 7% and 9% reduces the yield of whole polished rice by 3% and 8%, respectively [3]. Milling and polishing are also the most energy-intensive stages, accounting for 45–58% of total energy consumption in rice processing [4]. Excessive rice processing leads to substantial nutrient losses, particularly of dietary fiber, B vitamins, and minerals, which are transferred to the byproduct rice bran. Consequently, the nutritional content of polished white rice is reduced by more than 70% compared with brown rice [5,6]. This loss occurs primarily during milling. In the initial grinding stage, the strong shear action of the sand roller removes 37.3% of the bran layer and 10.9% of the germ, whereas subsequent iron roller milling removes 58.2% of the residual bran and 58.9% of the germ [7]. The combined action of silicon carbide and iron roller mills further exacerbates nutrient depletion, reducing fat, crude fiber, vitamin B_1_, and vitamin E contents by 48.6%, 60.0%, 59.4%, and 76.8%, respectively. Long-term consumption of excessively processed rice may contribute to “hidden hunger,” thereby increasing the risk of chronic diseases [8]. These findings underscore the importance of promoting and implementing moderate rice processing to improve product yield and enhance the edible utilization of food resources, thereby supporting food conservation, loss reduction, and food security.

In recent years, moderate processing has been widely promoted in the rice processing industry. It involves removing a limited portion of the indigestible and palatability-reducing outer layers while maximizing the retention of nutritional components, reducing food and energy losses, and meeting relevant national standards. However, rice produced through moderate processing differs noticeably in appearance and sensory attributes from fully polished rice. Additionally, the retention of the germ increases susceptibility to lipid oxidation and spoilage during storage because of its high fat content, thereby shortening shelf life. Contaminants such as heavy metals, pesticide residues, and mycotoxins are also more concentrated in the outer layers, potentially increasing food safety risks [9]. Therefore, achieving a balance among multiple quality attributes is critical in moderate rice processing. This article examines moderate processing technologies, analyzes associated quality changes, and reviews related processed products to provide a reference for improving and promoting moderate rice processing practices.

Previous studies have mostly focused on moderate processing techniques or single quality evaluation, lacking systematic and comprehensive discussions, and a comprehensive and objective evaluation of the advantages and disadvantages of moderately processed rice. This review discusses various nutrients and bioactive components in moderately processed rice, advocating for the consumption of moderately processed rice by people around the world; Analyzed the quality deterioration of precision rice during storage and proposed a rational solution; Described the process requirements for retaining native nu-trients during processing, providing direction for the research and development of pro-cessing equipment. At the same time, it has been clarified that the accumulation of heavy metals and pesticide residues are also key factors that deserve attention in moderate pro-cessing of rice. This article examines moderate processing technologies, analyzes associ-ated quality changes, and reviews related processed products to provide a reference for improving and promoting moderate rice processing practices.

## 2. The Principle of Milling Brown Rice

The cortical cells of brown rice exhibit a mesh-like structure with rectangular or elliptical shapes, and their distribution and density vary significantly among varieties. Japonica rice typically exhibits a denser, more uniform structure, with greater ductility and tensile strength. The tensile strength is higher in the longitudinal direction (Y) than in the radial direction (X). During milling, cortical cracking follows distinct patterns, with linear cracks along the Y direction and serrated cracks along the X direction [10,11]. Milling removes the bran layer from brown rice primarily through friction. This process includes four stages: initial sliding, surface bran layer friction, transition friction, and stable core friction [12]. Wear begins with friction between brown rice and the drum screen surface, followed by inter-grain friction that progressively removes the bran layer [13].

**Figure 2:**
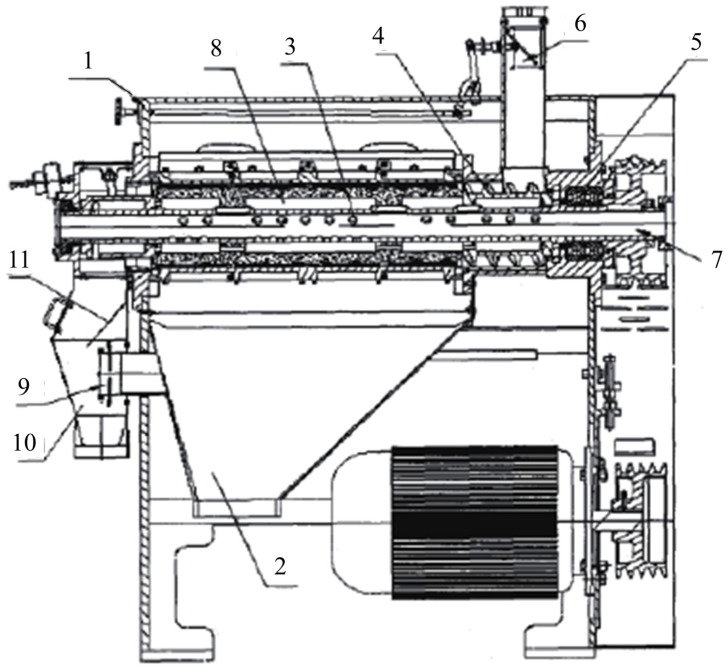
Schematic diagram of brown rice mill. 1. Rack; 2. Collect rice bran hopper; 3. Hollow shaft; 4. Spiral head; 5. Spindle sleeve; 6. Brown rice feed inlet; 7. Rice milling room; 8. Sand roller; 9. Rice cooling device; 10. Rice outlet; 11. Material guide plate.

The schematic diagram of the brown rice mill is shown in Figure 2. The bran layer is removed in a specific sequence: lateral, ventral, and then dorsal. In the milling chamber, brown rice grains in contact with the sieve interact mainly through the lateral surface, leading to preferential removal of the lateral bran layer. Removal efficiency varies with milling pressure. During milling, the outer gelatinous layer first undergoes longitudinal wear, followed by progressive peeling of the bran layer. This peeling occurs radially from pre-existing damaged regions as grains rotate and rub against each other. Due to variations in indentation and contact forces, bran layers in different regions are removed sequentially [14]. A common phenomenon in rice milling is the preferential orientation of grains, which leads to uneven initial damage [15]. Milling uniformity depends on the ability of grains to move from low-activity regions (“blind spots”) to the outer ring of the milling chamber. Promoting the exchange of axial and radial positions within the milling chamber improves the uniform removal of the bran layer. Greater frequency of axial–radial position exchange is associated with higher milling uniformity [16].

## 3. Characterization Methods for Processing Accuracy

Processing accuracy refers to the extent of residual rice germ, surface bran, and dorsal groove bran after milling. According to GB/T 1354-2018 [17], rice processing accuracy is classified as fine milling or moderate milling based on the residual bran level. Common characterization methods include weight-based, appearance-based, and component-based characterization.

### 3.1. Weight-Based Characterization

Weight-based characterization evaluates processing accuracy using the mass loss of rice grains before and after milling, expressed as DOM or the reduction rate:

DOM = (pre-milling mass − post-milling mass)/pre-milling mass × 100%.

A higher DOM indicates greater processing intensity. Brown rice has a DOM of 0%, whereas finely milled rice typically ranges from 12% to 15%, and moderately milled rice ranges from 8% to 12%. Increasing milling intensity reduces the retention of biological activity; hence, a DOM of approximately 9.5% has been suggested as optimal [18]. Structural heterogeneity within the rice grain also influences milling behavior. The dorsal bran layer has a thicker structure and stronger mechanical properties than other regions, resulting in its removal after the ventral bran layer. This sequential removal can increase endosperm loss in the ventral region and contribute to higher DOM values [19].

Artificial intelligence (AI) is increasingly applied to DOM analysis, integrating machine learning, deep learning, and spectroscopic techniques to improve speed and accuracy [20]. A hybrid approach that combines computer vision with fuzzy logic has been used to develop a fuzzy inference system for DOM analysis [21]. In addition, models based on the Inception-v3 architecture and residual networks (ResNet) have been integrated into an InceptionResNet Bayesian optimization algorithm, enabling more comprehensive feature extraction and accurate DOM prediction, with an average accuracy of at least 96.90% [22]. By integrating You Only Look Once version 8 (YOLOv8) with instance segmentation and oriented bounding box detection, real-time DOM analysis during milling has also been achieved [23]. Further improvements to YOLOv8, including the incorporation of dense blocks and optimized detection heads, have led to the development of the DSS-YOLOv8 model, which achieves processing speeds of up to 88.5 frames per second [24]. Moreover, a dual-branch spectral transformer network that combines hyperspectral imaging with deep learning has been proposed, achieving an accuracy of 97.54% [25].

### 3.2. Appearance-Based Characterization

Appearance-based characterization assesses processing accuracy by comparing changes in rice grain appearance before and after milling. Common indicators include bran retention and whiteness.

**Figure 3:**
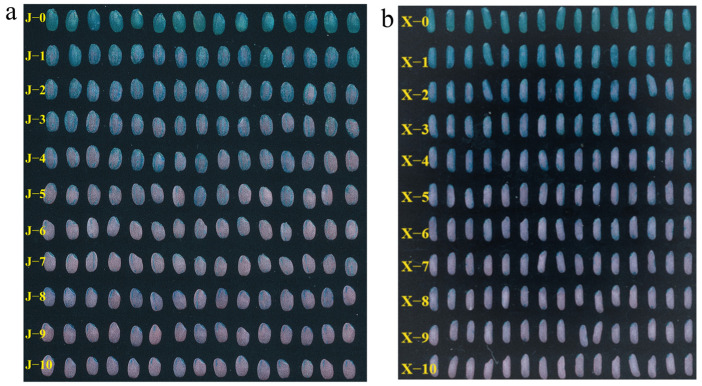
Appearance of Japonica and Indica Rice with Different Processing accuracies after Dyeing. (**a**) Appearance of japonica rice with different processing accuracies after dyeing [29]; (**b**) Appearance of indica rice with different processing accuracies after dyeing; J−0~J−10 DOM 0%~10% of japonica rice; X−0~X−10 DOM 0%~10% indica rice.

Bran retention is typically determined using staining methods. These methods exploit differences in dye affinity among the rice cortex, embryo, and endosperm using dyes such as eosin Y–methylene blue, magenta carbonate solution, or Sudan III ethanol solution. After soaking and rinsing, different grain regions exhibit distinct colors, which are evaluated manually or instrumentally against standard samples for processing accuracy (GB/T 5502-2025 [26]). The appearance of colored glutinous and indica rice with DOM values of 0–10% is shown in Figure 3. According to GB/T 1354-2018, bran retention in moderately milled rice ranges from 2.0% to 7.0%. Computer vision methods have been developed to compare images of milled and rough rice grains. These methods extract contour features and calculate the area, length, and width ratios to predict DOM. The reported accuracies are 95.63% for short-grain rice, 93.45% for long-grain rice, and 95.36% for medium-grain rice [27]. In addition, explainable machine learning approaches have been used to construct image-based DOM datasets through grain image acquisition and color processing, achieving an accuracy of 91.31% [28].

Whiteness refers to the brightness of the rice grain surface and can be measured using instruments such as colorimeters (L*, a*, b*) and whiteness meters. Brown rice shows a trend of yellow to white from the outside to the inside. During the grinding process, the outer yellow skin layer is gradually removed, and the white color of the endosperm is gradually exposed. The whiteness value gradually increases, and finally, the obtained rice whiteness is 42–47. Considering the balance of taste and quality, the whiteness of moderately processed rice should be 35–40. Specifically, brightness (L*) increases with the increase in DOM, while yellow (b*) and red (a*) decrease with the increase in DOM. Finally, when only the endosperm remains, the color value remains stable [30]. When DOM increases from 0% to 9%, whiteness, transmittance, and L value show a gradual increase trend, while a* and b* values gradually decrease to 180% and 58%, respectively. When DOM is between 9% and 15%, the endosperm is completely exposed, while a* and b* remain unchanged [31,32]. Using near-infrared reflectance spectroscopy, DOM, color parameters (L*, a*, b*), and whiteness values can be measured, with correlation models approaching a coefficient of 1 [33]. In addition, differences in bran thickness and milling behavior between colored and non-colored rice varieties affect processing efficiency, with colored rice typically requiring longer milling times to achieve comparable translucency [34].

### 3.3. Component-Based Characterization

There are significant differences in the nutritional composition and content among the seed coat, embryo, and endosperm of brown rice [35]. The contents of phosphorus, thiamine, fat, crude fiber, and related components gradually decrease with increasing DOM. When milling is excessive, the grain consists mainly of endosperm, and the levels of these components tend to stabilize. Therefore, chemical composition can be used to characterize processing accuracy. As the DOM increases, the contents of protein, fat, and total dietary fiber decrease, whereas total starch content increases, with more pronounced changes observed in fat and dietary fiber (*p* < 0.01). The contents of γ-globulin, vitamin B_1_, phenolic acids, and total flavonoids decrease to varying extents with increasing milling time [36]. The lipid content of brown rice is about 2.3%, and it gradually decreases with the increase in DOM. The lipid content of moderately processed rice decreased to 0.6%, and the lipid content of rice decreased to 0.3%, which is only half of that of moderately processed rice [37].

A comparison of the three methods for characterizing rice processing accuracy is presented in Table 1.

## 4. Correlation Between Moderate Processing and the Comprehensive Quality of Rice

Changes in processing accuracy alter the tissue structure of rice by progressively removing the bran and endosperm layers. These structural changes substantially affect rice composition and consequently influence sensory, nutritional, storage, and safety quality.

### 4.1. Moderate Processing and the Sensory Quality of Rice

In terms of flavor, increasing DOM gradually reduces the content of 2-acetyl-1-pyrroline, a key volatile aroma compound in fragrant rice, while the volatile profile changes nonlinearly with increasing milling time [38]. In mature barley rice, 14 key volatile flavor compounds, including alcohols, aldehydes, ketones, and esters, were identified, with relative odor activity values (ROAVs) ≥ 1. Among these compounds, seven decreased significantly as DOM increased [39]. Using untargeted metabolomics, 50 volatile compounds were identified in silk seedling rice (indica rice) across four processing levels, including 25 differential volatile compounds [40]. In addition, six aroma-active compounds were identified in japonica rice with different processing accuracies using gas chromatography–olfactometry and ROAV analysis. Rice with 6% DOM exhibited the highest abundance of volatile compounds and the most desirable aroma characteristics [41].

In terms of taste quality, milling promotes the formation of cracks on the rice grain surface, thereby progressively increasing water absorption during soaking and steaming. When part of the bran cell wall is retained, rice exhibits improved cooking and sensory properties [42]. This effect is attributed to the widespread distribution of thick cell walls and thermally stable starch granules within the bran layer. During cooking, channels formed in the bran layer facilitate water penetration, exerting limited effects on cooking properties, including optimal cooking time, water absorption, volume expansion, and total solid loss, while significantly affecting texture attributes such as hardness and adhesiveness, as well as peak viscosity [43]. When DOM exceeds 8.1%, the skin layer of brown rice is basically completely removed, and the water absorption rate, moisture distribution, and texture characteristics of the obtained rice gradually stabilize. Therefore, it is recommended that DOM 8.1% be used as the optimal processing accuracy for moderate milling [44]. The thickness of the rice endosperm layer is regulated by the *qDAT3.1*, *qDAT3.2*, and *qDAT7.1* genes, with the *qDAT7.1* allele significantly increasing endosperm thickness [45]. In addition, the morphology and crystal structure of rice change significantly across DOM levels ranging from 0% to 15% [46]. As DOM increases, the hardness and chewiness of cooked rice decrease, whereas cohesiveness and elasticity increase. Peak gelatinization viscosity also increases by 20.76% [47]. Increasing DOM significantly increases the amount of starch leached during cooking; however, the molecular size and chain-length distribution of the leached starch remain relatively unchanged. This process increases rice stickiness and contributes to a softer, glutinous texture [48]. Rice samples with different DOM values (0%, 3%, 6%, and 9%) also exhibit distinct retrogradation behaviors. Higher DOM is associated with a higher retrogradation index (ΔHr/ΔHg) during storage at 4 °C or −20 °C. Samples stored at −20 °C with lower DOM show greater structural contraction and more pores, accompanied by lower crystallinity and slower retrogradation rates [49].

### 4.2. Moderate Processing and Nutritional Quality of Rice

Milling can substantially reduce nutrient content, with micronutrients showing the greatest losses [50]. Compared with rice at 10% DOM (moderately processed rice), rice at 15% DOM (excessively processed rice) showed iron and zinc losses of 20.94–23%, whereas losses at 10% DOM were 10.87–12.9% [51]. In selenium-rich rice, selenium loss increased linearly with increasing DOM (R^2^ = 0.899, *p* < 0.01) [52]. After cooking rice with different DOM levels, bound VB_1_ content was significantly higher than free VB_1_ content. When DOM was below 8%, the increase in bound VB_1_ after cooking was inhibited, while thermal stability remained at approximately 80% and bioavailability at 60% [53]. This effect occurs because low DOM promotes the complete release of VB_1_ in the pregastric region, increasing its susceptibility to intestinal transformation and reentry into digestion. Therefore, maintaining DOM below 6.07% has been recommended [54]. The noncovalent affinity between VB_1_ and starch is stronger than that between VB_1_ and proteins or dietary fiber. Milling alters VB_1_ stability by changing the composition of matrix components that interact with VB_1_ [55]. Compared with brown rice, rice with 11% DOM showed significant reductions in vitamin E and phenolic contents, accompanied by an approximately 60% decrease in antioxidant activity, as measured by DPPH activity [56]. As average DOM increased from 0% to 2.67%, 7.25%, and 9.60%, total phenolic content decreased by 21.1%, 42.6%, and 55.6%, respectively, whereas cellular antioxidant activity decreased by 37.4%, 84.0%, and 92.8%, respectively. At 9.60% DOM, free ferulic acid and coumaric acid were no longer detectable in japonica rice [57].

Colored rice contains higher levels of total phenolic compounds and exhibits stronger antioxidant activity than conventional brown rice [58]. The content of free and bound phenolic compounds in the bran layer of black rice is higher than that of brown rice, and red rice exhibits higher DPPH and ABTS + antioxidant activity [59]. Black rice undergoes 0–60 s of grinding to obtain different levels of DOM, resulting in significant loss of nutrients. After 10 s of grinding, anthocyanins, gamma glutamylin, and alpha tocopherol decreased by 74.5%, 55.4%, and 70.3%, respectively. After 60 s of grinding, the phenol content and antioxidant activity significantly decreased, with antioxidant activity decreasing by 21.1 times [60,61]. When black rice reaches 6% DOM, the loss rates of anthocyanins, fat, ash, and phenolic compounds are 70%, 44%, 33%, and 31%, respectively. In contrast, the loss of protein and dietary fiber is relatively low, at 15% and 25% respectively [62]. The DOM of quinoa increased from 0% to 27%, while the total saponin content decreased by 41.8%, and the total phenolic and flavonoid contents decreased by 31.5% and 41.4%, respectively. The total antioxidant activity decreased by 40.7% [63].

### 4.3. Moderate Processing and Rice Storage Quality

Lower milling intensity helps reduce the loss of bioactive compounds; however, rice with lower DOM exhibits faster nutrient degradation, greater lipid oxidation, and lower pH during storage. Lipid oxidation is a major factor contributing to nutrient loss and is significantly negatively correlated with DOM (*p* < 0.01). Among the affected compounds, γ-aminobutyric acid (GABA) the greatest loss during lipid oxidation, followed by phenolics and vitamin B_1_ [64].

Auto-oxidation and photo-oxidation are the two primary pathways of lipid oxidation in rice [65]. A major characteristic of oxidation is the development of off-odors. Compounds such as 2-methylbutanal and furans contribute cocoa-like, nutty, and malty aromas and are considered key volatile compounds in rice. In contrast, butyraldehyde, hexanal, 2-hexenal, butyric acid, hexanoic acid, valeric acid, and heptanoic acid are recognized markers of rice spoilage [66]. During auto-oxidation, larger amounts of volatile compounds and oxidation products are generated, whereas light exposure further accelerates oxidation. Hexanal and 2-heptenal are the dominant volatile compounds under thermal conditions, whereas 2-heptenal and 1-octen-3-ol predominate under light irradiation, ultimately resulting in unpleasant odors [67]. Oxidation products derived from rice bran lipids can also induce protein oxidation, characterized by significant increases in protein carbonyl and tyrosine contents and a concomitant reduction in free thiol content [68]. Enhanced disulfide bond formation further promotes globulin aggregation and cross-linking, thereby reducing its foaming ability, foam stability, emulsifying activity, and emulsifying stability of globulin [69].

### 4.4. Moderate Processing and Rice Safety Quality

Common toxic heavy metals in rice include mercury (Hg), lead (Pb), chromium (Cr), cadmium (Cd), and arsenic (As) [70]. High-performance liquid chromatography–inductively coupled plasma mass spectrometry is commonly used to analyze inorganic arsenic (i-As), whereas inductively coupled plasma mass spectrometry is used to determine total As, Cd, Pb, and Hg contents [71]. Soil contamination and plant uptake are the primary pathways for heavy metal accumulation in rice [72]. Soil pollution assessments have shown relatively low levels of Cr and Pb contamination in paddy fields, whereas As contamination is more pronounced, potentially due to agricultural chemical use and irrigation with mine water [73]. Co-contamination with Pb and Cd in paddy soils poses a major threat to rice safety. Pb accumulates mainly in roots, with limited translocation to grains; however, Pb enhances Cd transport through vascular tissues, including internodes and nodes, resulting in a 54% increase in Cd accumulation. Therefore, Cd and Pb exhibit synergistic accumulation effects [74]. In areas near Dhaka, Bangladesh, industrial wastewater has been used for paddy irrigation, leading to extremely high concentrations of heavy metals, including Pb, Cd, Cr, nickel, and Hg, in both soil and irrigation water. In some cases, these concentrations exceed the safety thresholds established by the World Health Organization by 2- to 15-fold [75].

Field experiments have shown that foliar application of 2,3-dimercaptosuccinic acid (DMSA) during the flowering stage significantly reduces the contents of Cd, Pb, total As, and i-As in rice grains by 47.95%, 61.76%, 36.37%, and 51.24%, respectively, without affecting the concentrations of essential mineral nutrients (e.g., Mn, K, Mg, Ca, Fe, and Zn). This effect occurs because DMSA promotes the transport of heavy metals from roots and lower stems to leaves while inhibiting their translocation from leaves to panicles and grains [76]. Interactions between microorganisms and plants can also reduce the transfer of heavy metals from contaminated soil to rice. The mutant strain Δ*dr2577* of Deinococcus radiodurans effectively inhibits Cd and Pb transport in rice, reducing Cd contents in roots and shoots to 71.6% and 60.9% of control levels, respectively [77].

In addition, inoculation with native fungal biological agents significantly reduces As accumulation in rice by approximately 75% and moderately decreases Cd accumulation by 15–25% [78]. Cerium oxide and iron oxide nanoparticles have also been shown to promote rice growth, enhance antioxidant enzyme activity, and increase antioxidant compounds, including phenolics, flavonoids, and anthocyanins, while reducing oxidative stress and Pb uptake in rice [79].

Dietary exposure to heavy metals can adversely affect maternal health during pregnancy and may seriously compromise neonatal health [80]. Heavy metals are unevenly distributed within rice grains, with Pb concentrated mainly in the husk, whereas As, Cd, and Hg are more abundant in the bran layer. Milling can reduce heavy metal concentrations in rice; however, the removal efficiencies differ among metals, with Pb showing the highest removal rate and Cd the lowest. Milling technology has been shown to reduce Pb, As, and Cd concentrations to safer levels. After 15 s of milling, Pb was almost completely removed, whereas As content decreased by approximately 35%.

In contrast, because Cd is distributed more uniformly throughout the grain, milling has limited effects on Cd reduction [81]. As DOM increased from 0% to 10%, the concentrations of heavy metals, including As, Cd, and Hg, in japonica rice decreased progressively [29]. Although moderate processing improves nutrient utilization efficiency, toxic metal contamination must also be appropriately controlled. At 9% DOM, substantial Pb removal is achieved, whereas losses of beneficial elements such as Ca, Cu, Zn, and Se remain relatively low, providing a more balanced outcome between safety and nutritional quality [82].

Milling also reduces pesticide residues in rice. After brown rice was milled into polished rice, residues of pesticides such as fenpropathrin, fluorophenylamide, and tebuconazole decreased by 68.745–93.16%, 64.49–90.25%, and 69.74–92.58%, respectively [83]. The relationships among rice quality characteristics, processing accuracy, and optimal DOM are summarized in Table 2.

## 5. Moderate Processing Technologies and Equipment

The traditional rice processing process includes cleaning, husking, paddy separation, whitening, polishing, and color sorting. Among these steps, whitening and polishing are the primary processes that affect rice processing accuracy. Conventional rice processing typically involves 4–6 whitening stages and 2–3 polishing stages, which often result in excessive processing. To reduce overprocessing and improve control of processing accuracy, technological innovations have focused mainly on whitening and polishing techniques and related equipment.

### 5.1. Light Rolling and Loss Reduction Processes

Rice mills are commonly classified as vertical or horizontal according to their structure and as sand roller or iron roller mills according to roller material. Sand roller mills use hard and sharp abrasive particles on the surface of high-speed rollers to grind the outer layers of rice grains, causing the bran layer to fracture and detach, thereby whitening brown rice. In practice, the initial milling stage is generally designed to prioritize bran removal, creating initial wear regions on the grain surface and improving subsequent milling efficiency [84]. Iron roller mills rely on the relative motion between rice grains and the whitening chamber screen, together with friction among grains, to cause the bran layer to slide along the endosperm surface. This process breaks and removes the bran layer, thereby whitening the brown rice. In both types of mills, friction is the principal mechanism for bran removal. Collision, whitening pressure, rolling action, and axial conveying are the fundamental elements governing rice milling performance [85].

Using conventional friction-based rice milling equipment, processing intensity can be reduced by decreasing the rice loading volume in the whitening chamber and lowering the rotational speed. During operation, brown rice continuously enters the mill. As the filling level increases, newly added grains first occupy the outer ring before moving toward the inner region. The increased pressure and contact area within the milling zone subject the grains to more severe abrasion and increase the likelihood of convective movement [86]. In addition, reductions in local spatial porosity combined with increased external energy input intensify fluctuations in the particle displacement field [87]. Reducing the loading volume facilitates grain rotation, improves milling uniformity, and promotes lower weight loss during light milling. Lower milling speed also decreases collision energy, collision frequency, and energy transfer efficiency, thereby reducing grain wear [88]. The axial dispersion coefficient and average effective collision rate in vertical rice mills increase with the grain aspect ratio at lower rotational speeds but decrease at higher speeds [89]. Accordingly, the use of multistage intermittent milling systems, combined with optimization of the milling intensity or milling duration at each stage, enables more precise control of processing accuracy [90]. A drum speed of 1200 r/min and a rice loading capacity of 40% in a vertical rice mill have been reported to provide optimal overall performance for moderate processing [91].

**Figure 4:**
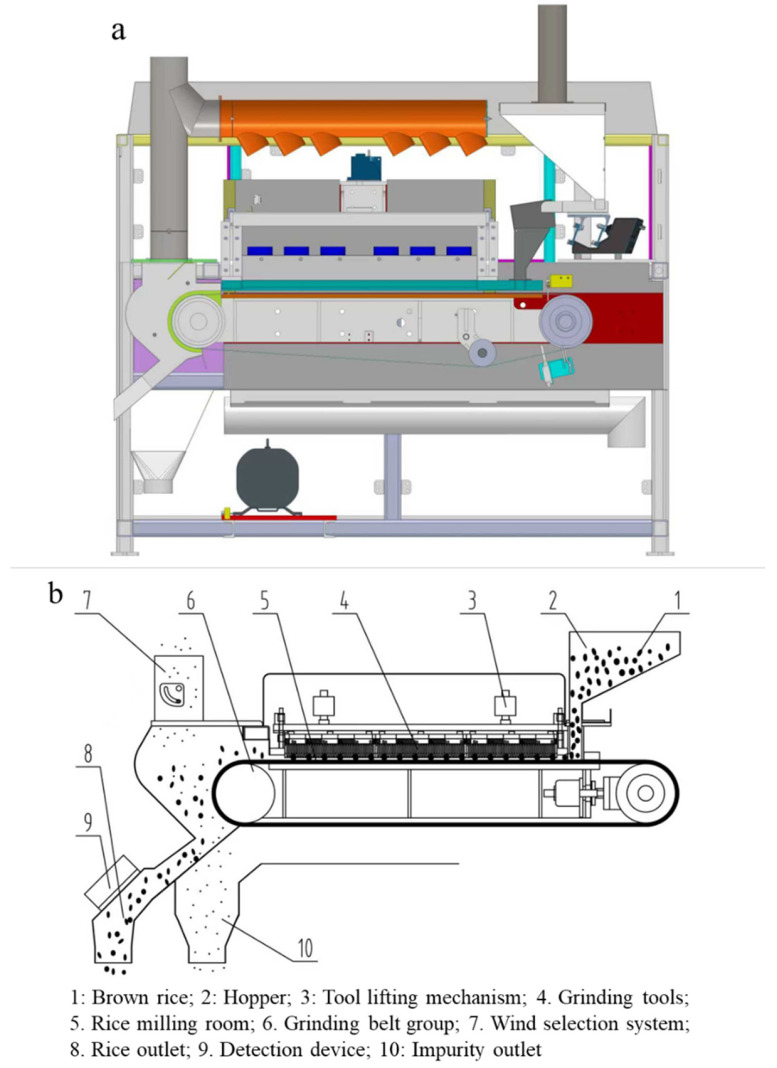
Special rice milling equipment for non pressure retained embryo rice. (**a**) Appearance diagram; (**b**) Internal structure diagram. The numbers 1–10 indicate the names of the structures of each part of the equipment.

Pressure-free rice milling equipment represents a third-generation energy-efficient milling technology. One type uses a layered flexible abrasive belt for milling. Brown rice enters the milling chamber and is uniformly milled through the high-speed linear velocity difference generated between the rice grains and the rotating abrasive belt. During this process, the grains rotate vertically, substantially reducing pressure within the whitening chamber, weakening the abrasive effect, lowering the breakage rate, and improving whitening uniformity. Another type is a specialized rice mill designed for embryo-retained rice (Figure 4). This system uses an abrasive belt and a multiunit elastic blade assembly as the primary milling components. Milling is achieved through the relative velocity difference between the linear motion of the abrasive belt and that of the elastic blade assembly. During operation, brown rice remains in a rolling state between the blades and the abrasive belt, producing a contour-like milling effect on the grain surface. The grains are effectively “wrapped” by the blade assembly, whereas the tapered structure protects most of the embryo, enabling relatively complete embryo retention. The embryo retention rate and embryo integrity both exceed 85%.

### 5.2. Low-Damage Polishing Process

Rice polishing is the final stage of rice milling. Its primary functions are to remove residual bran powder, smooth surface cracks, improve surface brightness, and increase grain compactness, thereby slowing oxidation, reducing microbial attachment, improving eating quality, extending storage life, and enhancing commercial value. However, conventional polishing equipment and processes are associated with several limitations, including high broken rice rates, nutrient loss, flavor deterioration, and increased energy consumption [92].

To meet the core objectives of damage reduction and quality improvement, rice polishing technology has evolved from emphasizing processing speed alone to balancing quality, efficiency, and energy consumption. Traditional polishing equipment commonly uses convex-rib polishing rollers, which can generate excessive localized pressure within the polishing chamber, leading to overpolishing. Newly developed flexible rice polishing machines incorporate elliptical ball-bar polishing rollers. This design increases the contact area between the polishing roller and rice grains, enhances inter-grain friction, and increases the frequency of collision and rolling, thereby improving polishing performance. In addition, the ball-bar structures are arranged in a staggered configuration to provide flexible buffering spaces, reducing excessive local pressure and enabling gentler polishing. The system is also equipped with an intelligent control platform that enables real-time monitoring of milling accuracy and supports intelligent process adjustment, thereby minimizing the trade-off between polishing smoothness and processing accuracy [87].

Flexible rice brushing machines use flexible materials, such as brushes, to gently scrape and brush rice grains. Their primary function is to remove bran powder from the grain surface through flexible contact, thereby reducing the broken rice rate, preserving the germ, and improving the nutritional quality of moderately processed rice. These machines typically adopt a vertical structure with top feeding and bottom discharge, allowing grains to move downward under gravity and minimizing the damage associated with forced mechanical conveying. During operation, cleaning is achieved through the coordinated action of flexible brush rollers and screen meshes. The paired brush rollers rotate in opposite directions, and the flexible bristles interact and rub against the rice grains, removing surface bran without generating the strong compressive forces associated with rigid rollers. Consequently, grain breakage is reduced. Compared with conventional polishing machines, flexible brushing systems provide greater protection of the rice germ and are particularly suitable for processing embryo-retained rice with high nutritional value. In addition, these systems use a dry-cleaning process that does not require water, thereby avoiding wastewater generation and associated treatment requirements [88].

## 6. Moderately Processed Rice Bran By-Products

### 6.1. Key Bioactive Substances in Rice Bran

After removing the outer shell of rice, it is ground and processed into rice and rice bran. Rice bran has always been used as a byproduct for feed or oil processing. In fact, rice bran is rich in dietary fiber, polysaccharides, and polyphenols, making it a valuable plant-based functional food resource [93]. Among them, rice bran polysaccharides have anti-inflammatory, antioxidant, immune-regulating, and gut microbiota-regulating effects, making them an emerging substance for delivery carriers, food additives, and bioactive ingredients [94]. Compared with rice, rice bran contains more nutrients and minerals. Vitamin E (tocopherol and tocotrienol) is 2.9 times that of rice, and minerals are 4.0 times that of rice. Ferulic acid shows the highest digestive stability and bioavailability (186–1159%) [95]. Black rice bran contains more gamma glutamylin (15.12 ± 0.03 mg/g), which has application prospects in preventing muscle atrophy [96].

By combining chemical and biological methods, 100% rice bran by-products are converted into bio-based products. Firstly, rice bran was further extracted with ethanol to produce 20.58% rice bran oil. The remaining 28.75% defatted rice bran after extraction was used as a carbon-rich substrate for microbial fermentation by Mediterranean hydrochloric acid bacteria. 12.75% was converted into polyhydroxybutyrate valerate, and the undigested 37.95% was processed into high-value cellulose, hemicellulose, and lignin. Rice bran was successfully converted into six industrial-value products [97].

### 6.2. Fermented Rice Bran

Most bioactive compounds in rice bran are bound by large molecular substances, resulting in low bioavailability Targeted microbial fermentation can degrade and modify complex macromolecules, releasing phenolic compounds, flavonoids, dietary fiber derivatives, and other new bioactive substances, effectively enhancing the antioxidant, anti-inflammatory, and metabolic regulatory effects of rice bran [98]. Solid state fermentation of rice bran by Aspergillus oryzae resulted in the identification of 448 bioactive compounds. The contents of shepherd’s purse acid, coumaric acid, vanillic acid, and ferulic acid increased significantly by 15–25 times, respectively. Fermented rice bran extract effectively enhanced antioxidant enzyme activity and reduced the levels of malondialdehyde (MDA), matrix metalloproteinase-1 (MMP-1), and interleukin-6 (IL-6) in HaCaT cells induced by UVB light aging [99].

## 7. Moderately Processed Rice Products

### 7.1. Lightly Milled Rice

Lightly milled rice is a moderately processed rice product with lower processing intensity than conventionally milled rice. Some studies classify rice with DOM values ranging from 0% to 9% as lightly milled rice [29]. Lightly milled rice retains part of the bran layer, where thick cell walls and thermally stable starch granules are widely distributed. During cooking, components released into the cooking liquid adhere to these structures, forming a reinforced surface film that improves the sensory properties of lightly milled rice. Presoaking can effectively increase water absorption and reduce grain hardness. In addition, steaming and boiling methods provide better retention of nutrients, food quality, sensory quality, and glycemic index stability [100].

GB/T 1354-2018 introduced the concept of “suitable for milling,” specifying that rice suitable for controlled milling should have a bran degree (BD) of 2–7%. As BD decreases from 10% to 0%, the bran layer is progressively disrupted, and starch granules flow outward and accumulate on the rice surface. Concurrently, defective kernels decrease from 33.15% to 1.00%, yellow kernels decrease from 4.50% to 0.05%, and fat, protein, and fiber contents decrease by 88.0%, 15.54%, and 34.48%, respectively. However, when BD remains between 5% and 10%, changes in whole-grain and broken-grain proportions are not significant. When BD ranges from 0% to 7%, rice hardness and cohesiveness show no significant differences, whereas elasticity and chewiness exhibit varying trends. A BD of 4% produces the highest taste value for raw rice, whereas the taste value of cooked rice declines more slowly when BD is maintained between 2% and 6%.

### 7.2. Embryo-Retained Rice

**Figure 5:**
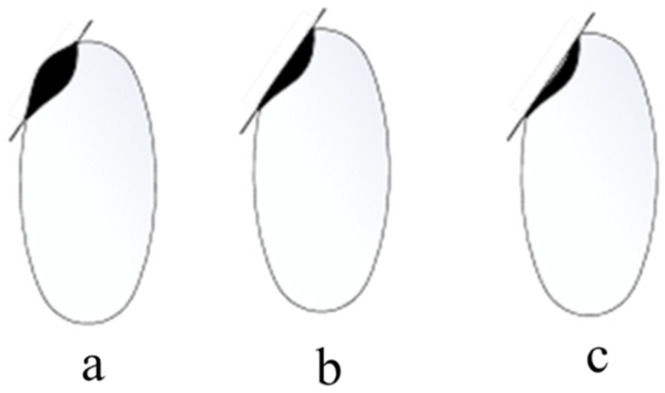
Schematic diagram of retained embryo rice grains. (**a**) Whole embryo rice grains; (**b**) Flat embryo rice grains; (**c**) Half embryo rice grain.

Embryo-retained rice, also referred to as embryo-retained rice, is a moderately processed rice product produced within the suitable milling range and characterized by the preservation of more than half of the intact rice germ during milling. Its classification is primarily based on the degree of germ retention. According to embryo integrity, embryo-retained rice is further categorized as whole-embryo rice (90–100% embryo retention; Figure 5a), flat-embryo rice (50–90% embryo retention; Figure 5b), and semiembryo rice (30–50% embryo retention; Figure 5c) (NY/T 4276-2023 [101]). The embryo retention rate should be ≥75% for indica rice and ≥80% for japonica rice according to GB/T 42227-2022 [102]. The rice germ is considered the most nutrient-rich component of the grain and contains approximately 66% of the total nutrients in rice. It is rich in GABA, proteins, B vitamins, vitamin E, and other bioactive compounds [103].

The degree of embryo retention directly influences lipid content, which subsequently affects the aroma profile of cooked rice, including compounds such as hexanal, nonanal, heptanal, octanal, decanal, (E)-2-decenal, (E,E)-2,4-decadienal, tetradecanal, acetyl compounds, and 2-pentylfuran. Therefore, maintaining embryo integrity is crucial for the quality of embryo-retained rice [104]. During embryo-retained rice processing, pretreatment with cellulase and xylanase can significantly improve milling efficiency by reducing abrasion resistance, thereby shortening milling time and increasing embryo retention. In addition, composite enzyme pretreatment disrupts the fiber matrix structure of the bran layer and significantly improves the cooking properties of embryo-retained rice [105]. An EfficientNet-B3-DAN model using an efficient network in online production efficiently and accurately identified embryo integrity, achieving an overall detection accuracy of 94.17% [106]. Furthermore, modifications to the Inception-v3 deep learning network, including the incorporation of mutual channel loss and mlpconv modules, improved recognition of embryo morphology and direction, resulting in a comprehensive recognition accuracy of 94.83% [107].

However, because embryo-retained rice preserves the germ, which contains high lipid content, its shelf life is relatively limited. The storage life of embryo-retained rice at room temperature is approximately 30 days, and lipid synthesis and metabolism are considered the primary pathways associated with quality deterioration during storage. To delay storage-related deterioration, polyethylene terephthalate/aluminum foil/polyethylene vacuum packaging has been applied to embryo-retained rice. This packaging method reduces lipid oxidative degradation, slows aldehyde accumulation, and minimizes undesirable odor changes during storage [108].

### 7.3. Moderately Processed Rice Foods

**Figure 6:**
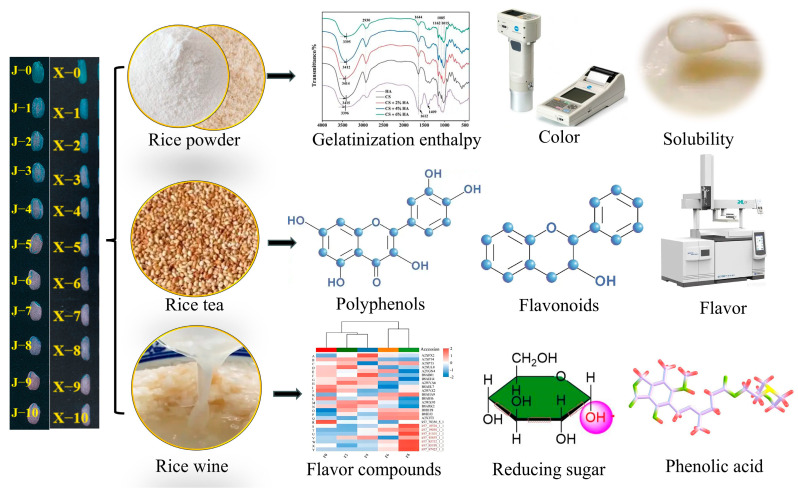
Application of rice with different processing accuracies.

Rice with different processing accuracies can be further processed into products such as rice noodles, rice porridge, puffed foods, rice tea, and rice wine (Figure 6).

The processing precision mainly affects the color, gelatinization degree and solubility of moderately processed Rice noodles when it is crushed to a certain size; The whole grain of rice is stir fried until it produces a unique aroma, while also adding active ingredients such as flavonoids and polyphenols. By using biological fermentation, the starch in rice is decomposed into active polysaccharides and phenolic acids, producing a unique aroma.

For rice noodles produced from rice with different DOM levels, the transition temperature does not change significantly, whereas gelatinization enthalpy increases with increasing DOM. The cooking characteristics of rice noodles are minimally affected by DOM; however, their color characteristics are influenced substantially. Rice noodles with higher DOM show higher brightness (L*) and lower yellowness (b*). In terms of texture, hardness and chewiness decrease significantly with increasing DOM [109]. In addition, rice noodles produced from rice with lower processing intensity exhibit better solubility than those produced from highly milled rice [110]. Rice porridge prepared from rice with different DOM levels also exhibits distinct quality characteristics. Milling removes the bran layer, promotes the migration of bound and free water during cooking, increases the water absorption index and swelling power of rice grains, and enhances starch leaching. These changes reduce the hardness and increase the viscosity of rice porridge. When the milling time reaches 80 s, the overall sensory quality of rice porridge is optimized [111].

Puffed foods prepared from japonica and indica rice with DOM values ranging from 0% to 8% show notable differences during extrusion processing. Amylose–lipid complexes formed during extrusion are negatively correlated with DOM. The expansion ratio, L* value, water absorption rate, and overall acceptability increase with increasing DOM, whereas hardness, water solubility, and bulk density decrease [112]. Rice tea produced from rice with DOM values ranging from 0% to 13% exhibits substantial reductions in bioactive compounds. Total phenolic content decreases by 48.12%, whereas total flavonoid and tannin contents also decline significantly. Rice tea prepared from rice with 2% DOM shows the smallest reduction in these compounds and the highest retention of aroma substances. Therefore, rice tea produced from rice with 2% DOM may improve safety and palatability while maximizing the retention of flavor compounds and nutrients [113].

Rice is the primary raw material used in yellow wine brewing. When DOM ranges from 5% to 15%, yellow wine exhibits relatively stable and superior quality, with improved clustering of flavor compounds and higher levels of alcohol, reducing sugars, and amino acid nitrogen [114]. Rice with DOM values ranging from 0% to 11% has also been used as a substrate for rice koji fermentation. Koji prepared from rice with 5–7% DOM showed high hydrolytic enzyme activity and contained abundant metabolites derived from hydrophilic compounds (e.g., sugars, sugar alcohols, organic acids, and phenolic acids) and lipid-derived compounds (e.g., fatty acids and lysophospholipids). In contrast, antioxidant secondary metabolites, including flavonoids and phenolic acids, were relatively higher in fermented koji prepared from 0% DOM rice. Overall, rice koji produced from substrates with 5–7% DOM was enriched in distinctive sensory, nutritional, and functional metabolites [115]. Defatted long-grain rice bran with DOM values of 2%, 4%, 6%, and 8% has been used to prepare gluten-free high-protein pancakes. Extended milling and defatting increased protein, essential amino acid, and ash content, and increased red/yellow color, while reducing fat content and altering protein secondary structure. The proportions of β-sheets, random coils, α-helices, and β-turns also increased. Pancakes prepared from defatted rice bran with 2% DOM exhibited superior texture and sensory properties [116,117].

## 8. Discussions

### 8.1. Conclusions

Compared with rice that only retains endosperm, moderately processed rice with DOM at 6–9% can retain 60–80% of dietary fiber and vitamin B1, while the content of unsaturated fat and minerals (such as magnesium and zinc) is significantly higher. That is to say, more than 66% of the nutrients in rice are concentrated in the germ and cortex, which only account for 2–3% of the mass. Therefore, in terms of nutrition, the key to moderate rice processing technology and equipment is to retain the germ. The representative product is retained embryo rice, which has been widely consumed in the field of infant and toddler complementary foods. In terms of safety, firstly, due to the fact that the embryo and cortex are located on the surface of rice grains, they are prone to accumulate heavy metals and chemical pesticides. Therefore, optimizing the planting environment and production methods is very important. Secondly, due to the high content of unsaturated fat in the embryo, it is prone to oxidation when exposed to air, which shortens the shelf life. Therefore, suitable packaging methods and storage environments need to be used to extend the shelf life. In terms of flavor and taste, rice with low processing precision has reduced flavor compounds, and the water absorption rate and amount during cooking have decreased, which affects the gelatinization of starch. As can be seen from the above, the determination of the optimal precision for moderate processing of rice requires the establishment of a multidimensional evaluation system including food, nutrition, and safety.

### 8.2. Study Limitations

The literature search in this review is extensive and structured, but different literature may have some differences in research conclusions due to different processing conditions and rice varieties, and there are also some differences in the determination of numerical values for processing accuracy. There is a lot of research on intelligent detection of processing accuracy using AI technology, but there is still no mature model for comprehensive quality analysis and evaluation, which requires a large amount of data analysis and import of different types of rice.

### 8.3. Lagging Standards and Standard Reconstruction

The ISO 7301 “Rice Specification” developed by the International Organization for Standardization (ISO) is the most core international standard in rice trade, with the latest revised version being ISO 7301:2021/Amd 1:2024-3-5 [118]. It categorizes the degree of rice mill-ing into three levels: Undermilled rice, Well milled rice, and Highly/Extra well milled rice, but international standards still use descriptive definitions.

In China, Current national rice standards are still primarily based on appearance-related indicators, such as processing grade and broken rice rate, while lacking quantitative requirements for nutrient retention. Consequently, enterprises that adopt moderate processing technologies to produce nutritionally enhanced rice products often face difficulties obtaining differentiated market recognition and pricing advantages. Therefore, dedicated standards for moderately processed rice products are urgently needed. Embryo-retained rice is a representative moderately processed product. Existing standards include GB/T 42227-2022 and NY/T 4276-2023. However, these standards mainly specify embryo retention rates and general milling requirements based on the characteristics of embryo-retained rice, without defining a precise range for moderate milling intensity. Therefore, standards specifically addressing moderate processing remain incomplete and require further refinement.

### 8.4. Onstraints on Technology Adoption

The initial investment required for intelligent production lines remains high. Complete AI-targeted rice milling systems are substantially more expensive than conventional milling equipment, limiting adoption by small- and medium-sized rice enterprises. In addition, moderate processing significantly increases the technical skill requirements for operators and demands interdisciplinary expertise. One potential solution is the development of a shared processing model, including the establishment of public moderate processing service platforms in major rice-producing regions to reduce barriers for small and medium-sized enterprises. At the same time, stronger collaboration between academic institutions and industry is needed to support specialized technical training and workforce development.

The existing rice mills, which mainly use grinding sand belts and multi unit elastic knife groups as the main grinding components, often have incomplete separation of bran and powder, making it difficult to produce continuously for a long time. The structure of the processing equipment still needs to be improved.

### 8.5. Development of Consumer Awareness

Many consumers still associate whiter rice with higher quality and remain insufficiently aware of the nutritional advantages of embryo-retained and suitably milled rice. Therefore, coordinated efforts across multiple sectors are needed to strengthen consumer education. One effective strategy is “nutrition visualization,” such as displaying comparative nutrient content for dietary fiber, vitamins, minerals, and bioactive compounds on product packaging, thereby enabling consumers to recognize and compare the nutritional value of moderately processed rice products. The successful development of the “Platinum Business Card” branding strategy for Jilin rice demonstrates that integrating regional branding with quality standards can effectively improve product recognition and premium market potential.

Overall, consumers’ perception of moderately processed rice is transitioning from “choosing based on appearance” to “paying for nutrition and actual taste”. Although tradi-tional concepts still have strong inertia, practical testing and popular science propaganda are driving a positive shift in this perception.

### 8.6. Future Direction: Whole-Industry-Chain Collaboration

The ultimate goal of precise and moderate processing should extend beyond optimization of the processing stage alone and encompass coordinated integration across the entire rice industry chain, from breeding to end-product utilization. At the upstream level, breeding programs can develop specialized rice varieties that are easier to mill, possess higher nutritional value, and exhibit stronger embryo stability to meet the requirements of moderate processing. Downstream, high-value rice milling byproducts, including rice bran, rice flour, and broken rice, can be further utilized in functional foods, starch sugars, pharmaceutical excipients, and related applications, thereby promoting cleaner and more efficient resource utilization. Only through coordinated integration of upstream and downstream sectors, together with stronger collaboration among industry, academia, and research institutions, can moderate rice processing evolve from a niche practice into a widely adopted industrial strategy.

The transition from conventional fine processing to precise and moderate processing represents not only an adjustment of processing parameters but also a fundamental shift in the understanding of rice value. The degree of rice processing reflects the industry’s definition of high-quality rice. As processing technologies achieve micrometer-level precision and AI systems enable real-time control of processing endpoints, future rice processing systems may simultaneously optimize sensory quality and health benefits. This integration of precision, nutrition, and intelligent control represents the core principle of moderate processing.

## Figures and Tables

**Table 1:** Comparison of methods for characterizing rice processing accuracy.

Methods		Principle	Instrumentation	Advantage	Limitation
Weight-based characterization		Quality loss rate	Analytical balance	Low cost, easy to operate	Due to factors such as grain shape, skin thickness, and processing parameters, the measured quality loss also includes some endosperm and broken rice.
Appearance-based characterization	Bran degree	Staining method	Manual or testing equipment	The instrument judgment method relies on computer image acquisition and processing technology, with high accuracy.	The subjectivity of manual judgment is strong, and the judgment results have high uncertainty
	Whiteness	White degree	Colorimeter or whiteness meter	Easy to operate	The numerical values are closely related to the variety, place of origin, cultivation method, chalkiness rate, etc. The detection results may have significant errors and relatively low reliability.
Component-based characterization		Standard curve	High performance liquid chromatography	None	In the early stage, it is necessary to establish a linear relationship between component content and processing accuracy. The detection sample size is large, the steps are cumbersome, and time-consuming.

**Table 2:** Correlation between moderate processing accuracy and the comprehensive quality of rice.

Quality Characteristic	Relationship with DOM	Optimal DOM
Flavor	The content of the key aroma compound 2-acetyl-1-pyrroline gradually decreases with increasing DOM, accompanied by changes in 25 differential volatile compounds [40].	Rice with 6% DOM shows the highest abundance of volatile compounds and the most desirable aroma characteristics [41].
Cookability and taste	When DOM exceeds 8.06%, the water absorption rate, moisture distribution, and texture properties of rice gradually stabilize. The hardness and chewiness of cooked rice decrease, whereas stickiness increases, resulting in a softer and more glutinous texture [43].	Rice with 8.06% DOM exhibits optimal water absorption, desirable sensory properties, and the slowest retrogradation rate [44].
Nutritional components	At DOM levels of 0–9.6%, vitamin B_1_, vitamin E, minerals, and total phenolic contents decrease by 20–55%, whereas antioxidant activity decreases by up to 92.8% [57].	DOM should be kept below 7.25% to maximize nutrient retention [62].
Heavy metal contamination	Cd, total As, and total Hg are concentrated mainly in the bran layer, whereas Cd is distributed more uniformly throughout the grain. Milling substantially reduces Pb and As contents, whereas Cd reduction is relatively limited [73].	At 9% DOM, heavy metal contents are substantially reduced, whereas losses of Ca, Cu, Zn, and Se remain relatively low, resulting in a better balance between safety and nutritional quality [82].

## Data Availability

No new data were created or analyzed in this study. Data sharing is not applicable to this article.
